# Spatiotemporal Mapping Techniques Show Clozapine Impairs Neurogenic and Myogenic Patterns of Activity in the Colon of the Rabbit in a Dose-Dependent Manner

**DOI:** 10.3389/fphar.2017.00209

**Published:** 2017-04-24

**Authors:** Susanna Every-Palmer, Roger G. Lentle, Gordon Reynolds, Corrin Hulls, Paul Chambers, Helen Dunn, Pete M. Ellis

**Affiliations:** ^1^Te Korowai Whāriki Central Regional Forensic Service, Capital and Coast District Health BoardWellington, New Zealand; ^2^Department of Psychological Medicine, University of OtagoWellington, New Zealand; ^3^Institute of Food, Nutrition and Human Health, Massey UniversityPalmerston North, New Zealand; ^4^Institute of Veterinary, Animal and Biomedical Sciences, Massey UniversityPalmerston North, New Zealand; ^5^Pharmacy Department, Capital and Coast District Health BoardWellington South, New Zealand

**Keywords:** clozapine, antipsychotic agents, gastrointestinal motility, gastrointestinal tract, constipation, ileus, spatiotemporal mapping

## Abstract

**Background:** Clozapine, an antipsychotic used in treatment-resistant schizophrenia, has adverse gastrointestinal effects with significant associated morbidity and mortality. However, its effects on defined patterns of colonic contractile activity have not been assessed.

**Method:** We used novel radial and longitudinal spatiotemporal mapping techniques, combined with and monitoring of ambient lumen pressure, in *ex vivo* preparations of triply and of singly haustrated portions of rabbit colon. We identified the contractile patterns of mass peristalses, fast phasic, and ripple contractions and directly qualified the effects of clozapine, at concentrations of 10 μmol/L, 20 μmol/L, and 30 μmol/L, and of norclozapine, the main metabolite of clozapine, on contractile patterns. The effects of carbachol, serotonin and naloxone on clozapine-exposed preparations were also determined. Tetradotoxin was used to distinguish neurogenic from myogenic contractions.

**Results:** At 10 μmol/L, clozapine temporarily abolished the longitudinal contractile components of mass peristalsis, which on return were significantly reduced in number and amplitude, as was maximal mass peristaltic pressure. These effects were reversed by carbachol (1 μmol/L) and to some extent by serotonin (15 μmol/L). At 10 μmol/L, myogenic ripple contractions were not affected. At 20 μmol/L, clozapine had a similar but more marked effect on mass peristalses with both longitudinal and radial components and corresponding maximal pressure greatly reduced. At 30 μmol/L, clozapine suppressed the radial and longitudinal components of mass peristalses for over 30 min, as well as ripple contractions. Similar dose-related effects were observed on addition of clozapine to the mid colon. At 20 μmol/L, norclozapine had opposite effects to those of clozapine, causing an increase in the frequency of mass peristalsis with slight increases in basal tone. These slightly augmented contractions were abolished on addition of clozapine. Concentrations of norclozapine below 20 μmol/L had no discernible effects.

**Conclusion:** Clozapine, but not norclozapine, has potent effects on the motility of the rabbit colon, inhibiting neurogenic contractions at lower concentrations and myogenic contractions at higher concentrations. This is the likely mechanism for the serious and life-threatening gastrointestinal complications seen in human clozapine-users. These effects appear to be mediated by cholinergic and serotonergic mechanisms. Spatiotemporal mapping is useful in directly assessing the effects of pharmaceuticals on particular patterns of gastrointestinal motility.

## Introduction

Whilst clozapine is uniquely effective for the treatment of otherwise treatment-resistant schizophrenia, it possesses a number of significant adverse effects including those on the gastrointestinal system. It has been shown to induce severe constipation, abdominal distension, ileus and intestinal obstruction and perforation, which can on occasion lead to death (Pelizza and La Pesa, 2007; Palmer et al., 2008; Hibbard et al., 2009; Every-Palmer et al., 2016). Further, therapeutic doses of clozapine are known to prolong colonic transit time (Baptista et al., 2015; Every-Palmer et al., 2016, 2017), an effect that is only partially ameliorated with assertive monitoring regimes and laxative treatment (Every-Palmer et al., 2017). While it is thought that disruption of colonic motility may affect between 50 and 80% of clozapine users (Baptista et al., 2015; Every-Palmer et al., 2016, 2017), with 30% experiencing constipation (Shirazi et al., 2016), and have a higher associated mortality rate than the better known adverse effect of agranulocytosis (Cohen et al., 2012), clozapine's gastrointestinal adverse effects remain poorly understood, under-recognized and inadequately researched (Flanagan and Ball, 2011; Every-Palmer et al., 2014).

It has been hypothesized that clozapine's effects on the gastrointestinal tract are mediated by blockade of cholinergic or serotonergic receptors (Palmer et al., 2008), with the drug known to have an anticholinergic effect on smooth muscle contraction (Ninan and Kulkarni, 1996) that is one tenth as potent as that of atropine (Ghosh, 2007). A number of pharmacokinetic studies on strips of uterine and intestinal smooth muscle have shown that clozapine can bind to and inhibit both serotonergic and cholinergic receptors (Kitazawa et al., 1998, 2000; Karaalp et al., 1999). Cholinergic activity is generally mediated in the colon via M_2_ and M_3_ receptors (Abrams et al., 2006) on neural and myogenic sites (Makhlouf and Murthy, 2006), and the blocking effect of clozapine on these receptors (Bolden et al., 1992; Li et al., 2005; Raedler, 2007) is reported to be proportional to the plasma concentrations of the drug (de Leon et al., 2003). Clozapine is also reported to inhibit contraction of smooth muscle in the rat jejunum by blocking serotonergic action at similarly distributed 5HT-3 and 5HT-7 receptors (McLean and Coupar, 1996).

It is possible that the potencies of the various pharmacological effects of clozapine on intestinal smooth muscle are augmented by derivatives that have been excreted into the lumen as well those that have been absorbed into the systemic circulation. Whilst clozapine is almost completely absorbed from the bowel, around 30% of the total dose enters the enterohepatic circulation as the metabolite norclozapine (Dain et al., 1997), which is has been previously reported to be pharmacologically active (Li et al., 2005).

Although there is detailed knowledge of the range of pharmacological activity of clozapine on various receptors, little is known of its effects on the various functional patterns of myogenic and neurogenic contractile activity exhibited by different segments of the gut. Most *in vitro* studies of human colonic motility have been performed on short strips of colon tissue (Huizinga et al., 1986; Huizinga and Waterfall, 1988) short segments of colon (Choe et al., 2010; Carbone et al., 2013) or surgically resected segments of colon in which motility is impaired (Dinning et al., 2016). The *in vivo* assay of specific patterns of human colonic motility has been complicated by technical problems incumbent in the methodology, notably by the shortcomings of colonic manometry (Dinning et al., 2014). We have not been able to find any reports of these techniques being used in clozapine-treated patients. Whilst both water-filled (Dinning et al., 2013), and fiberoptic catheters have been used to assess excursions in lumen pressure in human subjects (Dinning et al., 2014), they can identify only a limited number of the five principal types of neurogenic and myogenic contractile patterns that have been identified by spatiotemporal mapping of *ex vivo* segments of the colon in a range of different mammalian species (Lentle et al., 2008; Hennig et al., 2010; Chen et al., 2013; Costa et al., 2013; Dinning et al., 2014), those in the rabbit being typical (Lentle et al., 2008).

The haustrated colon of the rabbit exhibits four distinctive patterns of phasic contraction, two of which are inhibited by tetrodotoxin i.e., are neurogenic, and two of which are not i.e., are myogenic (Lentle et al., 2008). Both types of neurogenic contraction are propulsive. The first, mass peristalsis comprises an extensive region of sustained and coordinated contraction of circular and longitudinal muscles that propagates rapidly, either proximally or distally, at a rate of around 8–21 mm/s. The second, haustral progression, comprises a succession of local annular rings of circular contraction that propagate slowly, either proximally or distally, at a rate of 0.13 mm/s (Lentle et al., 2007). The two types of myogenic phasic contraction are thought to induce mixing. The first, ripple contractions, are short-lived local contractions of circular muscle that propagate a limited distance longitudinally at a speed of between 1 and 8 mm/s. The second, fast phasic contractions, are short-lived repetitive contractions chiefly of longitudinal muscle that propagate rapidly longitudinally at around 30 mm/s (Lentle et al., 2008). Apart from these four patterns of phasic contraction, the colon can also exhibit generalized long-lasting changes in tone (Tonini et al., 1982) i.e., a concerted and sustained contraction of the colonic musculature that reduces or increases the level of wall compliance and the capacity of the lumen (Lentle et al., 2008).

Preceding electrophysiological work based on the appearance of the changes in membrane potential that are associated with contraction (Sarna et al., 1981), and on episodes of elevation in lumen pressure (Dinning et al., 2013), identified colonic migrating motor complexes (CMMCs) which likely resulted from neurogenic mass peristalses with background higher frequency oscillation that likely resulted from myogenic ripple contractions. Hence these techniques could not readily distinguish radial from longitudinal muscle contraction, and hence the various known patterns of contractile activity exhibited by the colon, and therefor electrophysiological techniques are unlikely to be useful in detailed examination of the effects of pharmaceutical agents on these various patterns.

The current work assays the effect of clozapine on specific patterns of neurogenic and myogenic contractions in *ex vivo* preparations of rabbit colon maintained under a dynamic 2.5 cm hydrostatic head, the responses being quantified by longitudinal and diametric i.e radial longitudinal spatiotemporal mapping and variations in intraluminal pressure.

## Materials and methods

The effects of clozapine and norclozapine on spontaneous motility in the rabbit colon was determined principally in the proximal (triple haustrated) colon under *in vitro* conditions with a dynamic 2.5 cm hydrostatic head using a similar methodology to that used by Lentle et al. (2007). The effect of clozapine on the mid (singly haustrated) colon was also explored.

All procedures were approved by Massey University Animal Ethics Committee (MUAEC approval No. 15/115).

### Preparation of colonic segments

A total of 20 domesticated New Zealand white rabbits (either sex), of 2–3 kg body weight, were anesthetized in an induction chamber with 5% halothane in 33% oxygen and 66% nitrous oxide. They were subsequently maintained on 1.5% halothane in oxygen and nitrous oxide via a facemask attached to a Bain's circuit during the surgery. The proximal colon was then excised via a ventral midline abdominal incision and the rabbit was subsequently euthanased (Cohen et al., 2012) with intracardiac pentobarbitone (125 mg/kg).

The *taenia libra*, the wider of the three taeniae in the triply haustrated portion of each colon, was then dusted with carbon black in order to provide a series of randomly spaced markers from which to assess longitudinal movement as change in strain rate during contraction. The excised segment of proximal colon was then placed in oxygenated Earle's HEPES buffer solution (HBS) maintained at 37°C. A 7 cm segment of either the proximal (triply haustrated) or the mid (singly haustrated) colon was cannulated at the oral and aboral ends, and mounted in a heated organ bath containing HBS solution pH 7.35 with the following composition (in mM): NaCl 124.0, KCl 5.4, MgSO_4_ 0.8, NaH_2_PO_4_ 1.0, NaHCO_3_ 14.3, HEPES 10.0, CaCl_2_ 1.8, and glucose 5.0. The solution was continuously oxygenated (95% O_2_, 5% CO_2_) and kept at 37°C with a flow rate of 60 mL/min (300 mL total bath volume). The total volume of superperfusate within the recirculating system was 800 mL. The radial positions of the two cannulae were adjusted so that the ventral-most taenia libra (Barone et al., 1973) was positioned in the center of the video field with the adjacent intertaenial domains (i.e., the band of circular muscles lying between two taeniae) visible to each side of the colon.

The aboral cannula was attached to an L piece with a large internal diameter (14 mm) with a 50 mm vertical effluent arm which served to accommodate the temporary increase in the standing 2.5 cm hydrostatic head during contractions and allowed it to be dissipated by backflow, as no one-way valve was fitted. The orad end of the colonic segment was connected to a syringe pump that was used to introduce warmed Earles HEPES solution into the lumen of the colon. The lengthwise position of the proximal cannula within the organ bath was adjusted to prevent sagging of the length of colon within the bath.

### Drugs and dosage

Crystalline clozapine (Lipomed Arles Switzerland) was dissolved in a 1:1 mixture of PBS and DMSO at pH 7.2 to obtain the various concentrations and doses that were directly instilled into the organ bath (300 mL). Stock solutions of 1, 2, and 3 mg each in 2 mLs of PBS: DMSO were added when required to an organ bath volume of 300 mL, to yield ambient concentrations of 10, 20, and 30 μmol/L, respectively.

Similarly crystalline norclozapine 0.5 and 2 mg (Sigma-Aldrich) were each dissolved in 2 mLs of a 1:1 mixture of PBS and DMSO at pH 7.2. Stock solutions were added to the organ bath when required to yield ambient concentrations of 5 and 20 μmol/L, respectively.

Tetrodotoxin 0.5 mg (Alamone labs) was dissolved in 1 mL of PBS to give a concentration of 5 μmol/L when added to the bath. This was used to validate whether particular patterns of contraction were neurogenic. Note that a high concentration was used in view of the mass of colonic tissue and to ensure prompt penetration through the thickness of the colonic wall.

A volume of 30 μL of a 100 mM solution of carbachol (Sigma pharmaceuticals) in PBS was added as required to yield a bath concentration of 1 μmol/L. Similarly, 450 μL of a 10 mM solution of serotonin (Sigma-Aldrich) in PBS at pH 7.2 was added when required at a bath concentration of 15 μmol/L.

A volume of 250 μL of a 1.2 mM solution of naloxone hydrochloride (DBL Hospira Ltd, Australia) dissolved in a 1:1 mixture of PBS and DMSO at pH 7.2 was added when required to give bath concentrations of 1 μmol/L. Solvent controls were run at a concentration of 0.003% (42.66 mmol L^−1^) DMSO in the bath as this was the maximal following the addition of drugs.

### Experimental procedures

Variation in intraluminal pressure was used as a basis for quantification of drug effects whilst spatiotemporal mapping of longitudinal and circular muscle layers was used to determine changes in the sites and timings and coordination of the various types of contraction

#### Establishing the organ bath

Sufficient Earles HEPES solution was pumped into the distal end of the colon to fill the lumen and give a dynamic hydrostatic head of 2.5 cm of water. A closed preparation with a dynamic hydrostatic head was chosen on the grounds that it consistently generated cyclic mass peristalses without significant haustral progression, thus simplifying recognition and distinction of neurogenic from myogenic contractions. In all cases the circulation of the fluid through the organ bath was suspended immediately prior to the addition of each of the various pharmaceutical agents and resumed 2 min after.

Tetrodotoxin was added to a number of otherwise untreated preparations prior to the trial to verify that the repeating contractile episodes were indeed neurogenic contractions i.e., mass peristalses.

#### Pressure recordings

Intraluminal pressure was recorded on a Statham P23XL (Spectramed, Oxnard, CA) physiological pressure transducer connected to a DC Bioamplifier (Neomedix, Sydney, Australia) and Powerlab 8SP (AD Instruments, Sydney, Australia).

Data were downloaded into Labchart and digitized recordings were transcribed into Excel and Systat spreadsheets for subsequent analysis. The baseline pressures, the peak pressure and the peak durations at 20 and 50% of height and the frequencies of series of successive peaks were determined before and following the addition of the various doses of clozapine and compared by repeated measures ANOVA in SYSTAT v11. The rationale for measuring the duration of peak pressure at 50 and 20% of peak height, rather than the duration of the entire pressure pulse, was to avoid difficulties in distinguishing the points of commencement and completion of the pulse in lumen pressure from baseline variation. Hence, whilst the use of this technique rendered the measure of duration sensitive to differences in the gradients of the upslope and downslope, it enabled more reproducible quantification of duration. Both 50 and 20% peak height were used to ensure that major changes in slopes did not influence the outcome of comparisons.

#### Image acquisition and processing

A monochrome video camera (Leutron Vision LV-75 CE, Glattbrugg, Switzerland) with a zoom lens (Cosmicar 12.5–75 mm) was mounted 450 mm above the organ bath. The output from this unit was directly connected to a PC equipped with a frame grabber card (National Instruments IMAQ PCI-1409). Images of 752 × 400 pixels were captured at a rate of 15 frames per second and written to hard disk as uncompressed TIFF files. This procedure yielded the high quality images necessary for generating high-fidelity maps with one pixel corresponding to 0.1 mm.

Each image sequence, comprising of around 5,500 frames, was processed using a custom image processing program written in the Delphi language, which generated a number of maps showing the motility of the colon.

#### Diameter and radial maps

The technique used to generate diameter and radial maps (D & R maps) was an extension of that used by other researchers (Hennig et al., 1999). Each image was thresholded to separate the colonic segment from the background. The threshold level was set to the minimum value of the smoothed intensity histogram. The upper and lower edges of the outline of the colon were traced on the thresholded image using a simple search algorithm. A straight line was drawn through the midpoint of *taenia libre* along the long axis of the colon separating it into upper and lower halves corresponding to the two visible intertaenial domains; the position of this separating line being invariant between frames. The height of the pixel column between the separating line and the two traces gave the upper and lower R maps.

The R maps were compiled with the long axis of the colon displayed in the horizontal (x) direction and with successive scans stacked sequentially in the vertical (y) dimension i.e., with run time increasing downwards from the top of the map. Thus, each row of the R map corresponded to a single frame with the intensity of each map pixel at a given point along the length corresponding to a radius in mm at that point. A smaller radius was represented by a lighter pixel shade and a larger radius by a darker shade as shown on the corresponding scale. The upper R maps were used throughout this work.

#### Longitudinal maps

The longitudinal contraction and relaxation of taenia libre that accompanied mass peristalsis were determined using the mapping methods described by (Janssen and Lentle, 2013). This technique determined relative longitudinal movement by cross-correlation between successive frames based on the movement of surface textural patterns. These movements could then be used to derive longitudinal strain rates along the taenia, where the strain rate was the rate of change of strain (ε) given by:

$$\begin{array}{l} \varepsilon =\frac{\Delta \text{L}}{\text{L}} \end{array}$$

where L was the length of a segment of muscle and ΔL was the change of length to the segment due to muscle contraction.

The longitudinal distribution of strain rate was used to indicate regions of the *taenia libre* that were undergoing contraction or relaxation i.e. muscle motility. As the cannulated ends of the colonic segment were fixed, a contraction in one location required extension at some other point along its length. Negative strain rate values represented contraction and positive values relaxation. Mapping of the strain rate along the taenia for each frame yielded a longitudinal strain rate map (L map) with the same dimensions as the D and R maps. To generate a color map of strain rate values, black was used to represent no change in strain, yellow to indicate contraction and blue to indicate relaxation.

## Results

### Lumen pressure

#### At baseline

**Figure 1:**
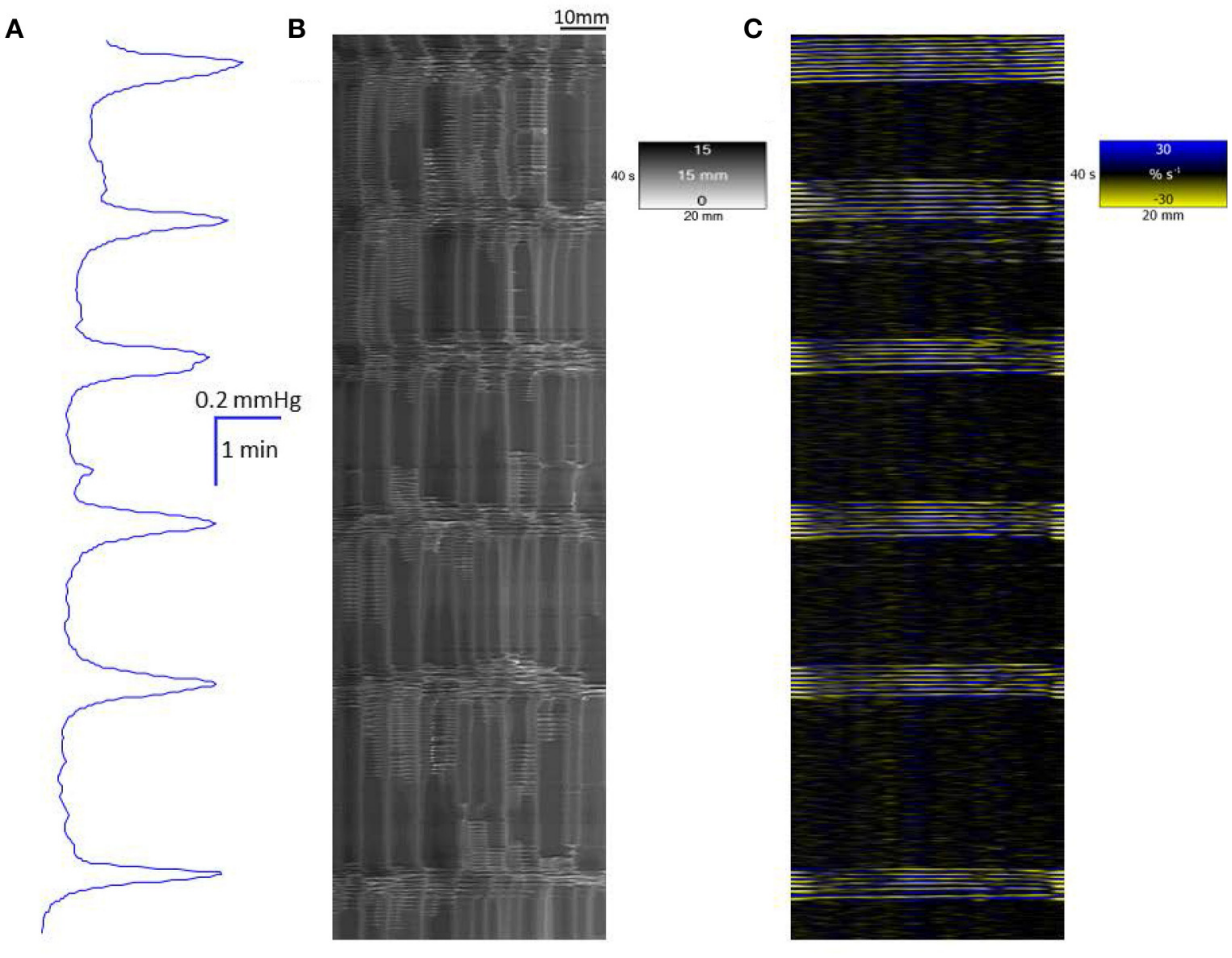
**Conformation of spontaneous regular mass peristalses in the ***ex vivo*** triply haustrated rabbit colon on R (B)** and L **(C)** spatiotemporal maps and concurrent variation in lumen pressure **(A)**. The proximal end of the colon is to the right and the distal end and hydrostatic head to the left. The varying numbers of high amplitude contractions of the longitudinal muscle of the taenia on the L map that comprise longitudinal mass peristaltic events are synchronous with bands of radial contractions on the R map and with the cyclic peaks in baseline pressure. Asynchonous radial contractions i.e., ripple contractions are also seen at various points along the colon, most frequently at the distal end where hydrostatic pressure is highest.

The mean baseline lumen pressure during spontaneous cyclic longitudinal and radial contractile activity in the proximal triply haustrated segment of the colon of a total of 20 rabbits was 0.28 ± 0.2 mm (mean ± sem) of mercury. Regular peaks in pressure, of a configuration typical of mass peristalsis also termed giant migrating contractions and CMMCs (Ehrlein et al., 1982; Sarna et al., 1984), occurred at a mean frequency of 0.57 ± 0.02 cpm. They each comprised a broad peak of mean duration of 3.5 s at 20% of maximum peak height with groups of high amplitude lower frequency pressure oscillations superimposed on the broader increase in baseline pressure (Figure 1).

#### Addition of tetrodotoxin

The addition of 0.5 mg of tetrodotoxin to the bath to give a concentration of 5.22 μmol/L (*n* = 3 preparations) caused the broad peaks in baseline pressure associated with mass peristalsis to cease, confirming that they were neurogenically mediated, whilst the lower amplitude, higher frequency oscillations in baseline pressure that were particularly evident during the broad peaks continued, confirming they were myogenic in origin.

#### Addition of clozapine

**Figure 2:**
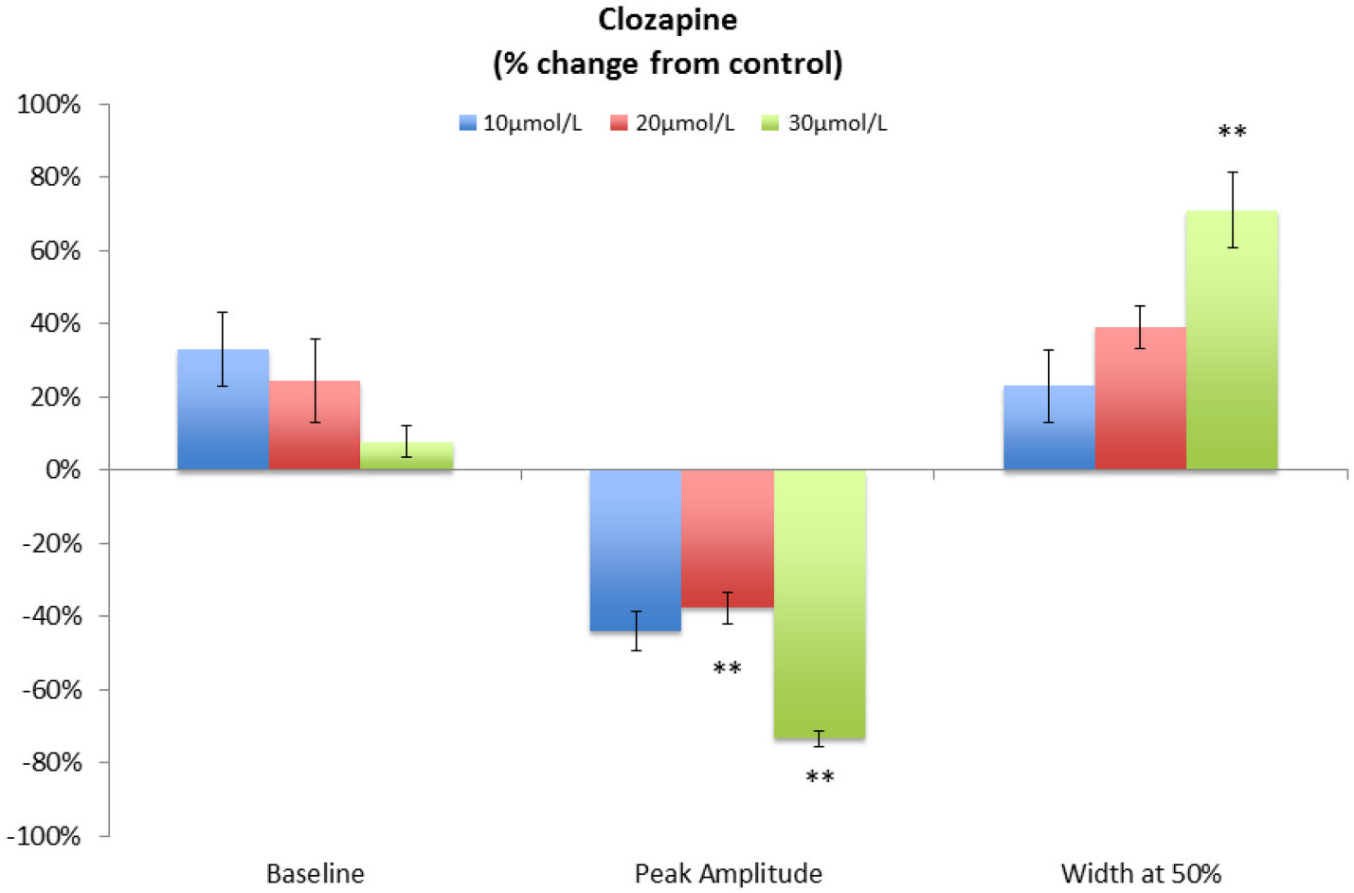
**Variation in pooled percentage changes in the characteristics of cyclic pressure peaks following addition of various doses of Clozapine**. Note that the duration is determined at the point where the pressure is at 50% of the peak height. This technique was used in order to avoid difficulties in detection of the point of commencement and completion of the pulse in lumen pressure. ^**^p < 0.01 (Significances of differences from control were determined using absolute values.

**Figure 3:**
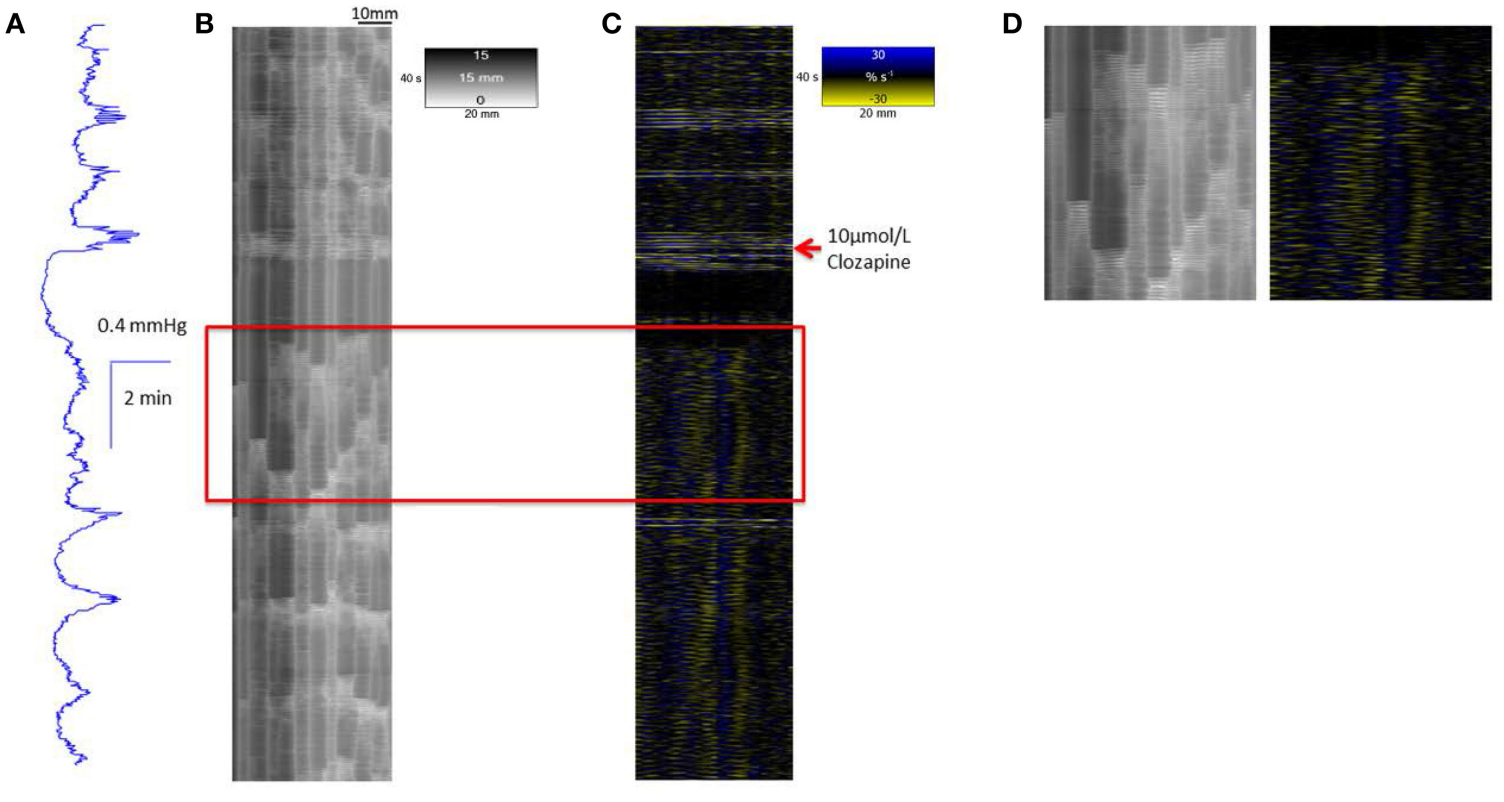
**Conformation of spontaneous regular mass peristalses in the ***ex vivo*** triply haustrated rabbit colon on R (B)** and L **(C)** spatiotemporal maps and concurrent variation in lumen pressure **(A)** following exposure to a concentration of 10 μmol/L clozapine. In each mass peristaltic event that occurred prior to the administration of clozapine the component increases in pressure from the groups of phasic longitudinal contractions and concurrent radial contractions are clearly shown along with interspersed ripple contractions. Following dosage with 10 μmol/L of clozapine pressure waves, longitudinal and accompanying radial contractions temporarily ceased whilst uncoordinated ripple contractions persisted (see magnified inset **D**). Subsequently, diminished pressure waves and accompanying barely visible longitudinal contractions that were coordinated with short weak radial contractions returned.

A concentration of 10 μmol/L clozapine (*n* = 3 preparations; **Figure 3A**) caused the regular broad pressure peaks accompanying of mass peristalsis to cease for around 3 min before returning with increased width and duration at 50% peak height (Figure 2). The numbers and amplitudes of the lower frequency, higher amplitude oscillations, that were particularly evident during the broad peaks, were reduced and temporally more dispersed during the 3 min period (Figure 3A). Subsequently cyclic peaks in pressure returned but with a reduced period of peaking in pressure (*n* = 3 preparations) compared with that prior to dosage (Figure 2).

**Figure 4:**
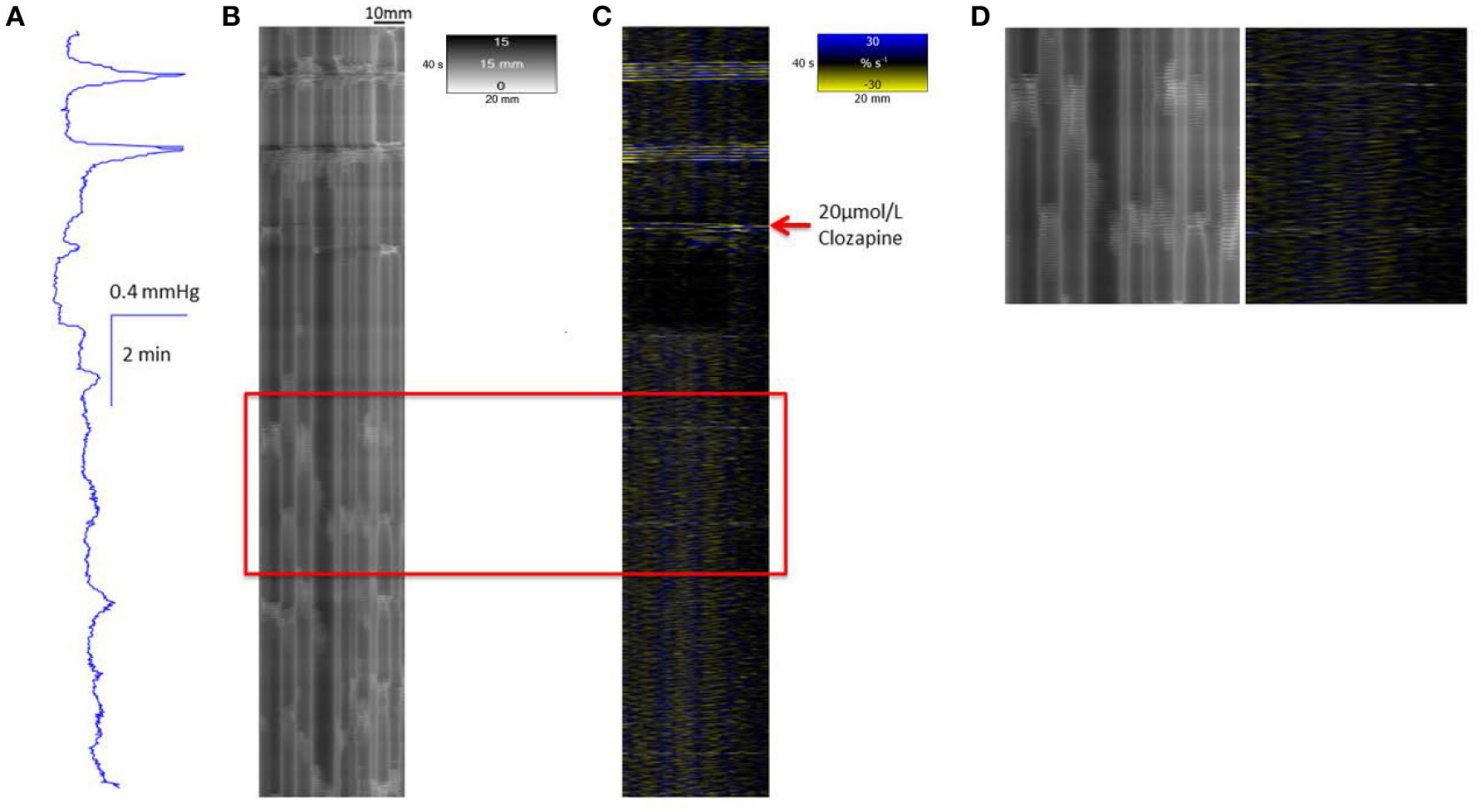
**Conformation of spontaneous regular mass peristalses in the ***ex vivo*** triply haustrated rabbit colon on R (B)** and L **(C)** spatiotemporal maps and concurrent variation in lumen pressure **(A)** following exposure to a concentration of 20 μmol/L Clozapine. As with 10 μmol/L clozapine, the coordinated radial contractions on the R map, the synchronous groups of longitudinal contractions on the L map and tall peaks in pressure are lost after dosage. The peaks in pressure are diminished in amplitude, as well as broader and irregular with only low amplitude peaks which also occur in the inter-peak interval. The groups of longitudinal contractions are abolished and single longitudinal contractions (Phase fast contractions) occur singly and irregularly. Intervening ripple contractions are reduced in number and distribution but some occur in concert with fast phasic contractions (see magnified inset **D**).

A concentration of 20 μmol/L clozapine (*n* = 3 preparations, Figure 4) initially abolished the regular peaks in pressure, which on their return were markedly reduced in overall amplitude, as were their component pressure oscillations. The durations of the peaks in pressure at 50% height were not significantly longer (*df* 1, 6 *F* = 5.29, *p* = 0.06) but their amplitudes were significantly lower (*df* 1, 8 *F* = 11.97 *p* = 0.009) than those before addition of clozapine (Figure 2).

The highest concentration of 30 μmol/L clozapine (*n* = 6 preparations) similarly caused the amplitudes of the peaks in baseline pressure to be significantly reduced (*df* 1, 4 *F* = 25.9 *p* = 0.007) and their duration significantly increased (*df*, 1, 4 *F* = 29.6, *p* = 0.006; Figure 2). Again the lower amplitude, higher frequency peaks that normally occurred during the broad peak were reduced and temporally more dispersed. In two preparations cyclic oscillations in pressure did not return after dosage, giving a persistent flat line configuration to the pressure trace.

In all cases the discernible effects of clozapine on pressure persisted for periods >15 min after washout. Similar effects were evident in preparations of the singly haustrated (mid) colon (*n* = 3). Hence, in summary, the peak pressure during mass peristalsis declined significantly with concentrations of clozapine of 20 μmol/L and above whilst the duration of the mass peristaltic pressure peak increased significantly. The baseline pressure also appeared also to decline, but this did not reach statistical significance (Figure 2).

#### Addition of carbachol, serotonin, or naloxone to the clozapine-exposed colon

**Figure 5:**
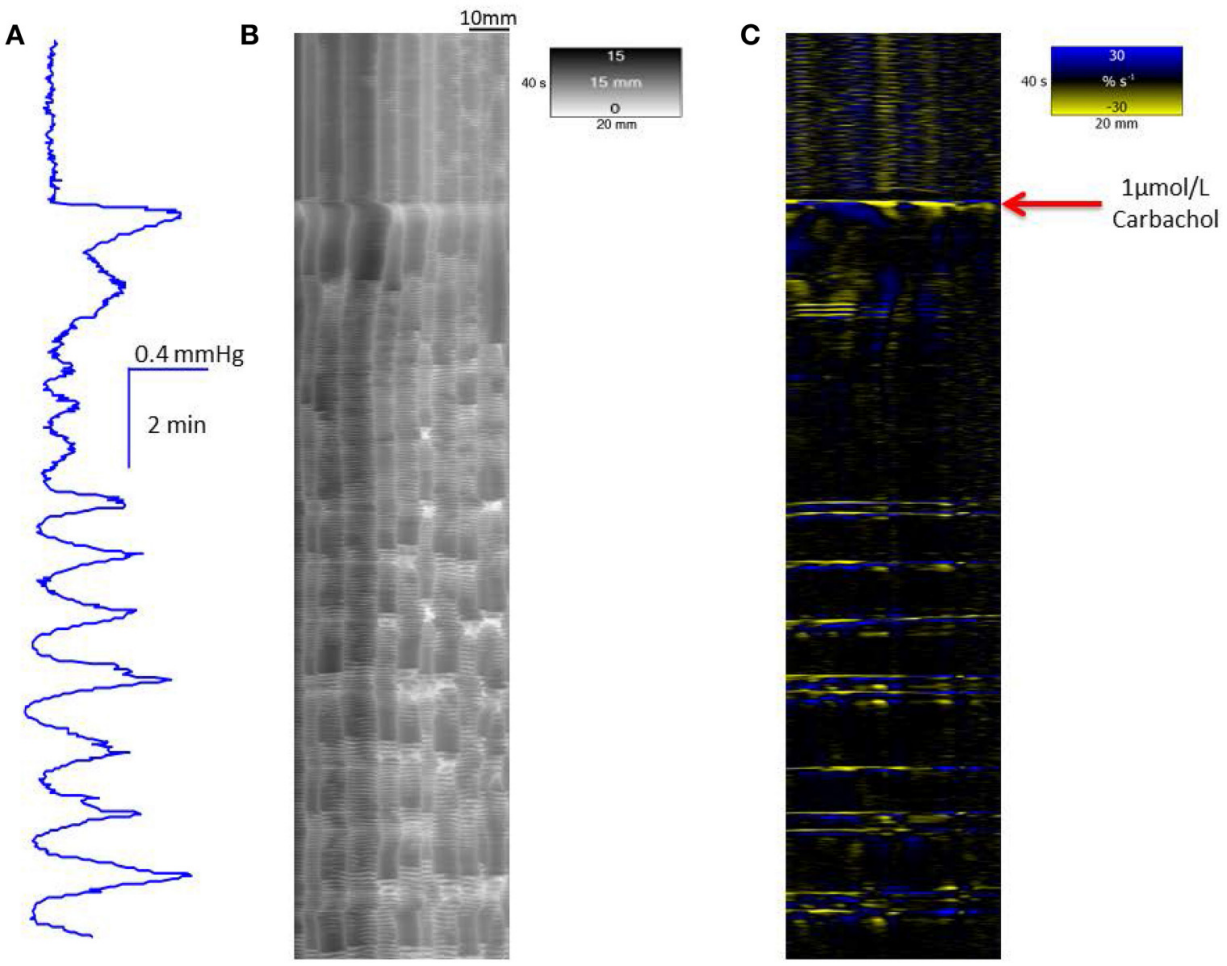
**Recovery of mass peristalses following dosage with 1 μmol/L carbachol in a segment of triply taeniated rabbit colon that has been dosed with clozapine at a concentration of 10 μmol/L**. The initial part of the curve taken after dosage with 10 μmol/L clozapine shows no grouped longitudinal contractile activity on the L map **(C)** and scant, randomly dispersed, ripple contractions on the R map **(B)**. Regular grouped longitudinal and synchronous radial contractions commence shortly after addition of 1 μmol/L carbachol and are coordinated with regular peaks in lumen pressure **(A)**. Supra-added peaks on the broader underlying peaks in pressure are in proportion to the number of longitudinal contractions.

**Figure 6:**
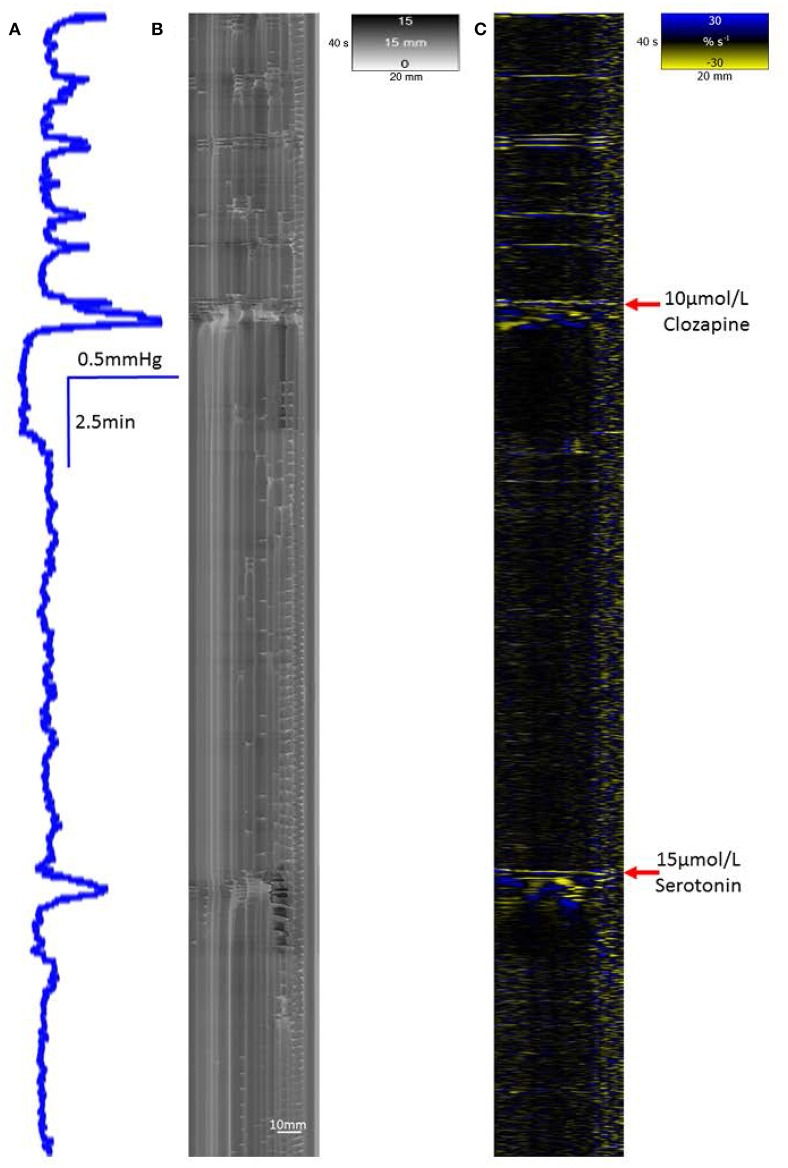
**Recovery of a single mass peristaltic contraction following addition of 15 μmol serotonin to a segment of triply taeniated rabbit colon that has been dosed with clozapine at a concentration of 10 μmol/L**. The L map following dosage with 10 μmol/L clozapine shows no grouped taenial longitudinal contractile activity on the L map **(C)** with scant randomly dispersed ripple contractions on the R map **(B)**. A single peak in lumen pressure **(A)** and associated single longitudinal and synchronous radial contraction occurr immeadiately after addition of 15 μmol/L serotonin to the bath with single a faint following organized contraction some 2 min later.

The addition of carbachol (1 μmol/L bath concentration) to preparations (n = 3) of the proximal colon that had been previously dosed with 10 μmol/L of clozapine caused an initial period of sustained elevation of pressure that preceded a subsequent return of cyclic peaks in baseline pressure associated with mass peristalsis (Figure 5). The addition of 15 μmol/L of serotonin to preparations that had been previously dosed with 10 μmol/L of clozapine caused an initial period of sustained elevation of pressure that preceded the return of one or two cyclic peaks in baseline pressure (Figure 6). The addition of naloxone (1 μmol/L bath concentration) to preparations (n = 3) of the proximal colon that had been previously dosed with 10 μmol/L of clozapine had no discernable effect in promoting the return of mass peristalsis.

#### Addition of norclozapine

**Figure 7:**
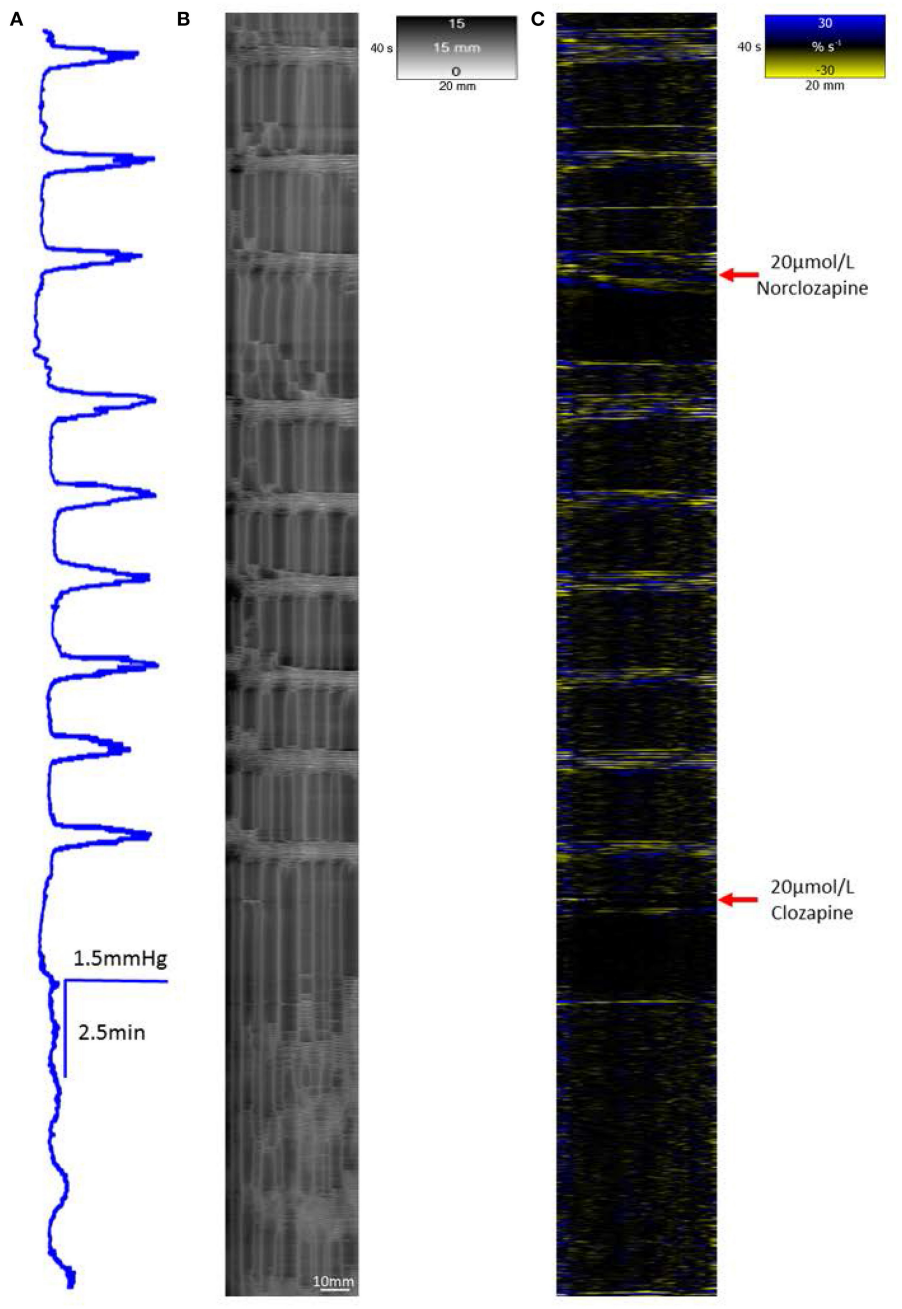
**The effect of 20 μmol/L Norclozapine and subsequent dosage with 20 μmol/L Clozapine on mass peristalsis in the proximal colon of the rabbit**. The baseline pressure and the maximum pressure changes associated with mass peristaltic events are both increased following dosage with 20 μmol/L norclozapine **(A)**. Both radial **(B)** and longitudinal **(C)** contractile activities associated with mass peristalsis are increased in frequency. Subsequent addition of 20 μmol/L clozapine markedly reduces cyclic changes in lumen pressure, eliminates longitudinal contractions and synchronous radial contraction while uncoordinated ripple contractions persist.

The addition of 20 μmol/L of norclozapine to preparations of the proximal colon (n = 3) caused the baseline pressure and overall cyclic peaks in pressure associated with mass peristalses to increase in a sustained manner (Figure 7).

### Spatiotemporal maps

#### At baseline

In the untreated, triply haustrated, proximal colon the radial and longitudinal components of regular mass peristalses were seen on R and L maps respectively (Figures 1B,C). On L type spatiotemporal maps (Figure 1C) each mass peristalsis comprised a sequence of 6–9 rapidly propagating longitudinal contractions that were broadly coincident with the high amplitude lower frequency components of the pressure traces. These events extended as near horizontal bands that indicated uniformly rapid propagation across the entire length of the preparation (Figure 1).

The synchronous R maps (Figure 1B) showed brief localized changes in diameter (mean colonic diameter 14.73 ± 0.62 mm) that were coordinated with longitudinal contraction and hence were neurogenic, as well as uncoordinated ripple contractions. Hence, a few ripple contractions were seen in the periods between mass peristalses (Figure 1B).

#### Addition of tetrodotoxin

The regular groups of longitudinal contractions on the L map that typified mass peristalsis were no longer evident after dosage with tetrodotoxin. However, infrequent single irregularly occurring longitudinal contractions that typify fast phasic contractions (Lentle et al., 2008) did occur. The short lived and localized diametric changes on the R maps that typified ripple contractions persisted but were temporally unsynchronized and dispersed along the length of the preparation.

#### Addition of clozapine

Exposure of *ex vivo* preparations of the triply taeniated colon to a concentration of 10 μmol/L clozapine initially abolished longitudinal contractions (Figure 3C) on the L map but not the radial constrictions on the R maps (Figures 3C,D). However, the latter events were no longer coordinated with longitudinal contractions and occurred irregularly at different sites along the preparation during this initial period i.e., were spontaneous myogenic ripple contractions rather than neurogenically mediated components of mass peristalses. Subsequently, the cyclic occurrence of regular groups of longitudinal contractions returned, though with fewer component contractions in each group (Figure 3C). These events were more difficult to discern on the L map but were coordinated with cyclic increases in pressure and increasingly coordinated with contractile events on the R maps (Figure 3B). Similar effects of this dose of clozapine were seen on mass peristalses in preparations of the singly taeniated (mid) colon.

Concentrations of 20 μmol/L of clozapine (Figure 4) had similar, though more marked, effects. Hence, after an initial period in which longitudinal contractions were abolished and contractions on R maps were no longer coordinated with them, there was a return on L maps of bands of longitudinal contraction that containing much reduced numbers of individual contractile events (Figures 4C,D) that were coordinated with cyclic changes in intraluminal pressure but had limited coordination with contractions on the R maps (Figures 4B,D).

Concentrations of 30 μmol/L of clozapine generally halted all longitudinal contractions on the L map and entirely suppressed the contractions on the R map along the length of the preparation for an extended period. Further, uncoordinated radial i.e., ripple contractions, generally did not return on the R map for over 30 min.

#### Addition of carbachol, serotonin or naloxone to the clozapine-exposed colon

The addition of carbachol at a concentration of 1 μmol/L to preparations that had been previously exposed to 10 μmol/L of clozapine (*n* = 3 preparations) caused an initial generalized circular contraction on the R map concurrent with a period of intense longitudinal contraction on the L map. This was followed by restitution of concerted longitudinal contraction and accompanying radial contractions consistent with the return of mass peristalses (Figure 5). This reversal did not occur in preparations that had been previously exposed to higher doses of clozapine.

The addition of serotonin in a concentration of 15 μmol/L to preparations that had been previously exposed to 10 μmol/L of clozapine (*n* = 3 preparations) caused an initial generalized circular contraction on the R map and a brief intense longitudinal contraction on the L map, which was followed by a limited number of concerted longitudinal and circular contractions (Figure 6).

The addition of naloxone to a bath concentration 1 μmol/L did not restore coordinated mass peristaltic contractions in preparations (*n* = 3 preparations) that had been previously treated with 10 μmol/L of clozapine.

#### Addition of norclozapine

The addition of 20 μmol/L of norclozapine to preparations of the triply taeniated colon (*n* = 3 preparations) caused the frequencies of the longitudinal contractions on the L map (Figure 7C) and the coordinated radial contractions on the R map (Figures 7B,C) to increase in frequency (Figure 7A). Subsequent addition of 10 μmol/L of clozapine caused longitudinal contractions to cease and loss of coordination with radial activity on the R map.

Addition of DMSO alone to a bath concentration of 0.003% (42.66 mmol) had no effect either on the amplitude, frequency or duration of cyclic changes in lumen pressure nor on the rhythmic characteristics of mass peristalses, ripple contractions, or fast phasic contractions.

## Discussion

### Clozapine had profound effects on the rabbit colon

We found that in the rabbit colon, clozapine had an immediate and significant effect on gastrointestinal motility. Neurogenic contractions i.e., the coordinated busts of longitudinal and circular contractions that occur during mass peristalsis, were impaired at all tested clozapine concentrations. Myogenic contractions in circular muscle were impaired at the higher tested clozapine concentrations.

Longitudinal muscle, which contracts during mass peristalsis and fast phasic contractions (Lentle et al., 2008), and the coordination of this activity with radial contractions, is more sensitive to clozapine than circular muscle. This suggests that clozapine had a greater effect on the neural components of the myenteric and submucosal plexi than on myogenic elements (Spencer et al., 2016). Given that similar lumen pressure events that are consistent with mass peristalsis and with uncoordinated radial i.e., ripple contractions are also reported in the human colon, (Holzknecht, 1909; Narducci et al., 1987; Dinning et al., 2014) it is likely that clozapine also exerts these effects on human colonic motility.

### Norclozapine did not decrease colonic activity, and may increase it

The results showing that *N*-desmethyclozapine (norclozapine), a hepatic metabolite of clozapine (Pirmohamed et al., 1995), promotes rather than interferes with colonic motility, was somewhat surprising given that norclozapine had previously been speculated to contribute to the drug's effects on gastrointestinal motility. Bailey et al. (2015) found clozapine-treated patients using laxatives had on average 29% higher norclozapine concentrations (mean = 0.337 mg/l; *SD* = 0.19) than those who did not use laxatives (mean = 0.269 mg/l; *SD* = 0.18); and hypothesized norclozapine's antagonistic properties at the M3 receptor could be promoting gastrointestinal hypomotility. Although norclozapine, like its parent drug, is indeed an M3 antagonist, it is also a potent M1 receptor agonist (Weiner et al., 2004), a molecular property not shared with any other antipsychotic. Li (Li et al., 2005) found norclozapine increased cortical acetylcholine release in the CNS, an effect that was completely inhibited by the M1-preferring antagonist, telenzepine. We consider it likely that it is these M1 agonistic properties that account for the difference in norclozapine and clozapine's effects on colonic motility.

While this needs further study, it is possible the processes of hepatic detoxification and subsequent biliary excretion (Park et al., 2015) of norclozapine serves to decrease any local adverse effects on colonic motility via the mucosa although, dose for dose at 20 μmol/L, norclozapine did not appear to ameliorate the effects of subsequent addition of clozapine. Hence plasma levels of clozapine are likely to be correlated with the degree of receptor blockade (de Leon et al., 2003).

### Clozapine's gastrointestinal effects can be mediated *in vivo* by cholinergic and serotergic agonists, but not opioid agonists

Clozapine is known to act at opioid receptors. In animal models, clozapine induces a potent anti-nociceptive effect in a dose-dependent manner with ED50 of 8.7 mg/kg (Schreiber et al., 1999). This effect is antagonized by the non-selective opioid antagonist naloxone (*p* < 0.05), implying an opioid mechanism of action is involved in clozapine-induced anti-nociception. Norclozapine has also been reported to act as a opioid receptor agonist (Onali and Olianas, 2007; Olianas et al., 2009). Activation of neural mu and kappa subtypes of opioid receptors have been reported to inhibit neurally mediated contraction of both circular and longitudinal muscle in the ileum of the guinea pig (Kosterlitz and Waterfield, 1972) and inhibit ileal peristalsis *in vitro* (Kromer et al., 1980) and opioids like morphine are potently constipating (Frantzides et al., 1992). Consequently it seems reasonable to hypothesize that opioid-like effects may contribute to clozapine induced gastrointestinal hypomotility and that agents which combat opioid-induced constipation may be he useful in clozapine-treated patients (Holzer et al., 2009).

However, our work showed that addition of 1 μmol/L of naloxone, a dose in excess of that necessary to reverse the effect of morphine on peristaltic spike potentials (Russell et al., 1982), failed to reinstate coordinated circular and longitudinal colonic contractions following exposure to 10 μmol/L clozapine.

The addition of the cholinergic agonist carbachol however, *did* restore coordinated circular and longitudinal colonic contractions, although not in the preparations that had received higher doses of clozapine. These findings fit in with earlier work showing that clozapine shifts the response curve of rat anococcygeal muscles to acetyl choline to the right (Ninan and Kulkarni, 1996).

Similarly, the addition of 15 μmol/L serotonin briefly restored coordinated radial and longitudinal contractions to preparations exposed to 10 μmol/L of clozapine, but did not reverse the effects of higher doses, the brevity of the intestinal contractile response being similar to that reported in the small intestine of the rabbit (Salvador et al., 2000).

Together, these results suggest that the colonic effects of clozapine are mediated by cholinergic and serotonergic receptors, presumably via M1-M_3_ (Eglen et al., 1996) and 5HT-3 and 5HT-7 receptors (McLean and Coupar, 1996) respectively. In respect of the latter, it is noteworthy that both serotonin 2C receptors and acetyl choline M3 receptors are known to act via identical G proteins, effectors and second messengers (Makhlouf and Murthy, 2006).

### Effect of DMSO

The results showing that concentrations of DMSO of 0.003% had no effects on cyclic changes in lumen pressure or on the characteristics of spatiotemporal maps are in line with previous work showing no effect of DMSO 0.1% (v/v) on tone or rhythm of *ex vivo* preparations of longitudinal muscle from rat ileum (Jeong et al., 2009), circular and longitudinal muscle of mouse colon (Wang et al., 2008) or longitudinal muscle from the rat colon (Zhang et al., 2010; Choi et al., 2011).

### Limitations

The limitations of the study include the difficulties in relating the concentration of clozapine in a water bath acting on rabbit colon to the concentration of clozapine in plasma and the human colon. Interference with neurogenic contractions i.e., mass peristalses, occurred over a clozapine dose range of 10 to 30 μmol/L. If we discount interspecies differences and relate the concentrations of clozapine in the organ bath to those in plasma, the bath concentrations are within an order of magnitude of plasma levels reported in patients receiving therapeutic doses of clozapine. Plasma samples from large scale therapeutic drug monitoring studies of 26,796 patients maintained on clozapine showed plasma concentrations were generally below 1 mg/l but were around 2 mg/l (i.e., 6.1 μmol/L) in 0.4% of subjects and as high as 2.55 mg (7.8 μmol/L) and 4.95 mg/l (15.2 μmol/L) in a few isolated cases (Couchman et al., 2010).

A second limitation was that the colonic segments studied were the haustrated rabbit colon rather than the haustrated human colon, although those of the two species are morphologically and physiologically similar (Lentle et al., 2008).

### Implications for the use of clozapine in human subjects

Our *ex vivo* findings in rabbits are entirely consistent with *in vivo* research showing that clozapine impairs motility in the human gastrointestinal tract, sometimes termed “clozapine-induced gastrointestinal hypomotility” or CIGH (Palmer et al., 2008; Flanagan and Ball, 2011; Taylor et al., 2015; Every-Palmer et al., 2016; West et al., 2017). CIGH causes prolonged colonic transit (Baptista et al., 2015; Every-Palmer et al., 2016), with an array of clinical manifestations from constipation (Shirazi et al., 2016) to life-threatening conditions such as paralytic ileus (Nielsen and Meyer, 2012) and toxic megacolon (Palmer et al., 2008). Our work in identifying the likely underlying pharmacological mechanisms, leads us to recommend avoiding additional anticholinergic and antiserotenergic medication when using clozapine, using the lowest effective dose, and assertive management of slow transit. Cholinergic and serotenergic agents require further investigation for the treatment of CIGH, but opioid antagonists like methylnaltrexone are unlikely to be effective. Pending the elucidation of more specific pharmacological strategies, the use of laxatives has been shown to be helpful. For example, using the “Porirua Protocol,” which encompasses monitoring and prophylactic laxative use in clozapine-treated patients, significantly improves colonic transit times and, over time, reduces serious sequelae (Every-Palmer et al., 2017).

### Conclusion

The results show that the effects of therapeutic agents with complex pharmacology on functional patterns of motility can be quantified in *ex vivo* segments of the gastrointestinal tract using the technique of spatiotemporal analyses to resolve the activity radial and longitudinal components of the various colonic contractions (Lentle et al., 2007). Also, that the judicious use of specific agonists and antagonists can elucidate which of the various pharmacological effects influence a particular functional pattern of motility.

Overall, clozapine had profound and persisting effects on the haustrated rabbit colon, inhibiting neurogenic contractions at all tested concentrations, and myogenic contractions at higher concentrations. These effects were specific to clozapine, and were not shared by its principle metabolite, norclozapine. Similar effects are predicted in the human colon, with clozapine's retardation of colonic contractile activity being the likely mechanism for the serious and life-threatening gastrointestinal complications that have been reported in human clozapine-users. However, spatiotemporal mapping also showed clozapine's inhibition of gastrointestinal motility could be reversed by cholinergic and serotonergic agents, although not opioid agonists.

These findings suggest cholinergic agents and/or serotonergic agents could potentially be useful agents in relieving colonic and other gastrointestinal side effects of clozapine, and this warrants further investigation.

## Author contributions

Conception and design of work: SE, RL, PE, and HD. Acquisition and analysis of data: RL, GR, CH, and PC. Interpretation of data: All authors. Drafting, revision, and approval of manuscript: All authors. Agreement to be accountable for all aspects of presented work: All authors.

## Funding

This work was supported by a University of Otago Research Grant 112005.01.R.RP. The funders had no role in the study design, data collection and analysis, decision to publish, or preparation of the manuscript.

### Conflict of interest statement

The authors declare that the research was conducted in the absence of any commercial or financial relationships that could be construed as a potential conflict of interest.
